# The duplicitous nature of ACE2 in COVID-19 disease EBioMedicine (invited commentary)

**DOI:** 10.1016/j.ebiom.2021.103356

**Published:** 2021-04-25

**Authors:** Samuel N. Heyman, Safa Kinaneh, Zaid Abassi

**Affiliations:** a Department of Medicine, Hadassah Hebrew University Hospital, Mt. Scopus, Jerusalem, Herzog Medical Center, Jerusalem, Israel; b Department of Physiology and Biophysics, Rappaport Faculty of Medicine, Technion-Israel Institute of Technology, Haifa, Israel; c Department of Laboratory Medicine, Rambam Health Care Campus, Haifa Israel 31096

Our understanding of the renin-angiotensin-system (RAS) has been revolutionized over the last 20 years with the discovery of angiotensin converting enzyme (ACE)2 and its principal product – angiotensin (Ang)-(1–7) [1]. ACE2 is a cell-membrane-bound protease, also appearing as a soluble form in the circulation, which converts Ang II to Ang-(1-7) [2,3]. Ang-(1–7) binds to Mas receptor (MasR) and angiotensin AT2-receptors (AT2R) and induces vasorelaxation through nitric oxide [1,2]. It also suppresses inflammation, oxidative stress, apoptosis, fibrosis and coagulation [1,2]. This tissue-protective arm of RAS is essential in counterbalancing the devastating effects of the RAS pressor axis, mediated by Ang II and AT1 receptor (AT1R), that exerts vasoconstriction and induces inflammation, apoptosis, fibrosis, coagulation and renal salt and fluid retention [1,[3], [4], [5]]. Thus, homeostasis requires balanced opposing actions of the pressor and depressor-protective arms of RAS. Indeed, altered generation of Ang-(1–7) has been associated with hypertension and tissue damage, leading in particular to renal and cardiac fibrosis and dysfunction [1,3,5]. Moreover, the induction of ACE2 or the administration of Ang-(1–7)- mimetics that activate MasR have recently been proposed in the management of various systemic and organ disorders [5].

Additional convertases such as circulating furin and transmembrane protease serine (TMPRSS2) strengthen viral attachment to membranal ACE2 by modification and cleavage of the S1/S2 spike proteins [6]. Thus, primary viral invasion likely occurs in epithelial cells expressing ACE2, such as the nasal mucosa, alveolar epithelial cells, pulmonary macrophages and the gut mucosa. Viral dissemination following replication in target homing tissues conceivably also requires ACE2 expression in the vasculature and various tissues such as the myocardium and renal tubular cells [1,3]. It has been suggested that enhanced expression of ACE2 increases susceptibility to SARS-CoV-2 infection [7]. Indeed, Genetic factors and male gender, as well as acquired conditions, such as air pollution and chronic lung disease, diabetes, heart failure, and the use of ACE inhibitors, all leading to enhanced expression of ACE2 in target organs, are likely associated with predisposition to SARS-CoV-2 infection [3].

Viral attachment to membranal ACE2 is followed by viral internalization together with ACE2 and ACE2 degradation, leading to depletion of membranal ACE2 [5]. Additionally, activation of ADAM-17 (shedase), triggered by Ang II, further reduces cell-bound ACE2 [8]. This leads to depletion of ACE2 and Ang-(1–7) at the tissue level, altering the balance between the pressor and depressor axes of RAS, in favour of the ACE/Ang II/AT1R pathway [5]. This is likely manifested by the clinical characteristics of COVID-19 disease, namely profound inflammation, oxidative stress, tissue injury, multi-organ dysfunction and coagulopathy [3,5]. A logical interventional approach might therefore be the restoration of balanced RAS by the stimulation of the ACE-2/Ang-(1–7)/MasR axis, in parallel with the inhibition of AT1R activation [5].

Taken together, high expression of membranal ACE2 facilitates viral homing, but its loss by degradation and shedding leads to Ang-(1–7) depletion, promoting the devastating clinical manifestations of COVID-19 disease [5]. Yet, many parts are still missing in the puzzle of SARS-CoV-2 and ACE2/Ang-(1–7) interactions.

For that reason, the study of Nikiforuk et al., published in *EBioMedicine* [9] is important, providing new perspectives, by looking at the association of SARS-CoV-2 positivity and the transcription of ACE2 and TMPRSS2 in nasopharyngeal samples, comparing 212 SARS-CoV-2–positive patients and 212 SARS-CoV-2-negative matched controls. The major findings reported are that among SARS-CoV-2 carriers, the transcription of membrane-bound ACE2 was proportional to viral replication, as opposed to an inverse association of viral load and the transcription of the ACE2 isoform lacking the trans-membrane domain. These observational findings are interesting, but cautious interpretation is needed due to present methodological limitations. Firstly, gene transcription in samples obtained by swabs or washout may not reflect true tissue expression, as it is affected by the composition of nasopharyngeal excretion and by tissue damage and turnover. Furthermore, it is believed that circulating ACE2 (lacking the transmembrane domain) is generated from cell-membrane expressed ACE2, shed by ADAM-17 and other proteases [8], a conversion that is accelerated during SARS-CoV-2 infection. Nikiforuk et al. checked de novo synthesis of mRNA putatively encoding truncated ACE2, using two distinct primers that span different sites along the *ACE2* transcript. It is important to consider the existence of alternatively spliced isoforms (Fig. 1) and to note that the detection of the two primer products does not necessarily indicate the existence of an independent formation of soluble ACE2 at the protein level. Thus, we believe the findings reported by Nikiforuk et al. and their conclusions will require further confirmation, looking at ACE2 protein abundance and not just transcription. This would revolutionize current concepts and may suggest an active mechanism of preventing viral invasion by using a synthesized form of circulating ACE2 to compete with cell-bound isoforms. Meanwhile, a more solid finding provided by Nikiforuk et al. is the positive association between viral load and ACE2 transcription in SARS-CoV-2 positive carriers, that underscores the importance of ACE2 expression in SARS-CoV-2 homing and infectivity.

**Fig. 1:**
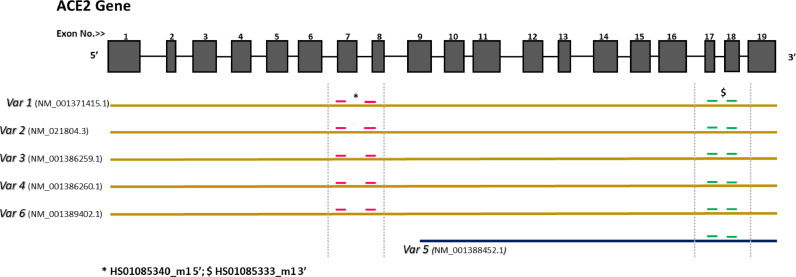
Homo sapiens angiotensin converting enzyme 2 (ACE2) - transcript variants. Fig. 1:*ACE2* is composed of 19 exons, which are transcribed into at least six transcript variants; five of them with high homology (in brown) and one with shorter length (shown in blue). This isoform misses the sequence that encodes the active soluble part of the protein. Red dashes refer to primers (HS01085340_m1) that spans the sequence encoding full-length transcripts (transmembrane + catalytic sites). Green dashes refer to another primer pair (HS01085333_m1) that spans the sequence that encodes all transcript variants, including the short isoform. In brackets are accession numbers of each transcript variant.

## Contributors

All authors contributed to conceptualization, writing, reviewing, editing and have read and agreed to the published version of the manuscript.

## Declaration of Competing Interest

The authors declare no conflict of interest.

## References

[1] Santos R.A.S., Oudit G.Y., Verano-Braga T., Canta G., Steckelings U.M., Bader M (2019). The renin-angiotensin system: going beyond the classical paradigms. American journal of physiology. Heart Circul Physiol.

[2] Sampaio W.O., dos Santos R.A.S., Faria-Silva R., Machado L.T.D., Schiffrin E.L., Touyz R.M. (2007). Angiotensin-(1-7) through receptor Mas mediates endothelial nitric oxide synthase activation via Akt-dependent pathways. Hypertension.

[3] Gheblawi M., Wang K., Viveiros A., Nguyen Q., Zhong J.C., Turner A.J., Raizada M.K., Grant M.B., Oudit G.Y. (2020). Angiotensin-Converting Enzyme 2: SARS-CoV-2 Receptor and Regulator of the Renin-Angiotensin system: celebrating the 20th anniversary of the discovery of ACE2. Circ Res.

[4] Abassi Z., Higazi A.A.R., Kinaneh S., Armaly Z., Skorecki K., Heyman S.N.ACE2 (2020). COVID-19 infection, inflammation, and coagulopathy: missing pieces in the puzzle. Front Physiol.

[5] Heyman S.N., Walther T., Abassi Z. (2021). Angiotensin-(1-7)—A Potential Remedy for AKI: insights derived from the COVID-19 pandemic. J Clin Med.

[6] Millet J.K., Whittaker G.R. (2015). Host cell proteases: critical determinants of coronavirus tropism and pathogenesis. Virus Res.

[7] Hofmann H., Geier M., Marzi A., Krumbiegel M., Peipp M., Fey G.H., Gramberg T., Pohlmann S. (2004). Susceptibility to SARS coronavirus S protein-driven infection correlates with expression of angiotensin converting enzyme 2 and infection can be blocked by soluble receptor. Biochem Biophys Res Commun.

[8] Patel V.B., Clarke N., Wang Z., Fan D., Parajuli N., Basu R., Putko B., Kassiri Z., Turner A.J., Oudit G.Y. (2014). Angiotensin II induced proteolytic cleavage of myocardial ACE2 is mediated by TACE/ADAM-17: a positive feedback mechanism in the RAS. J Mol Cell Cardiol.

[9] Nikiforuk A.M., Kuchinski K.S., Twa D.D.W., Lukac C.D., Sbihi H., Basham C.A., Steidl C., Prystajecky N.A., Jassem A.N., Krajden M., Patrick D.M., Sekirov I. (2021). The contrasting role of Nasopharyngeal Angiotensin Converting Enzyme 2 (ACE2) expression in SARS-CoV-2 infection: a cross-sectional study of people tested for COVID-19 in British Columbia. E Bio Med.

